# Removal of inorganic mercury from aquatic environments by multi-walled carbon nanotubes

**DOI:** 10.1186/s40201-015-0209-8

**Published:** 2015-07-28

**Authors:** Kamyar Yaghmaeian, Reza Khosravi Mashizi, Simin Nasseri, Amir Hossein Mahvi, Mahmood Alimohammadi, Shahrokh Nazmara

**Affiliations:** Department of Environmental Health Engineering, School of Public Health, Tehran University of Medical Sciences, Tehran, Iran; Center for Water Quality Research (CWQR), Institute for Environmental Research (IER), Tehran University of Medical Sciences, Tehran, Iran

**Keywords:** Adsorption, Aqueous solutions, Heavy metals, Nano material

## Abstract

**Background:**

Mercury is considered as a toxic heavy metal in aquatic environments due to accumulation in bodies of living organisms. Exposure to mercury may lead to different toxic effects in humans including damages to kidneys and nervous system.

**Materials and methods:**

Multi-walled carbon nanotubes (MWCNTs) were selected as sorbent to remove mercury from aqueous solution using batch technique. ICP instrument was used to determine the amount of mercury in solution. Moreover, pH, contact time and initial concentration of mercury were studied to determine the influence of these parameters on the adsorption conditions.

**Results:**

Results indicate that the adsorption strongly depended on pH and the best pH for adsorption is about 7. The rate of adsorption process initially was rapid but it was gradually reduced with increasing of contact time and reached the equilibrium after 120 min. In addition, more than 85 % of initial concentration of 0.1 mg/l was removed at 0.5 g/l concentration of sorbent and contact time of 120 min. Meanwhile, the adsorption process followed the pseudo second-order model and the adsorption isotherms could be described by both the Freundlich and the Langmuir models.

**Conclusion:**

This study showed that MWCNTs can effectively remove inorganic mercury from aqueous solutions as adsorbent.

## Background

Water resource pollution with industrial effluents is known as a serious environmental problem, nowadays [1].Of these, scientists have focused mostly on the presence of mercury due to its bioaccumulation in organisms, toxic effects and persistence in environment [2, 3]. In addition, it should be noted that mercury has been widely used in various industrial fields as an element including chlor-alkali, pharmaceutical, producing barometer and thermometer, mining, dental practices. It is proved that high concentrations of mercury are released constantly from the mentioned industries to the environment [4, 5].

From the toxicological point of view, the level of toxicity of mercury is highly related to its chemical form [6]. To put it another way, mercury transforms biologically, physically, and chemically through its cycle in nature, which results in the formation of various forms of mercury. Organic mercury is the most toxic among these forms [7]. Mercury is mostly in its inorganic (Hg^+2^) or methylmercury forms in aquatic environments [6]. However, at the presence of specific kind of bacteria the inorganic form of mercury transforms to methylmercury which is highly toxic for human and other organisms at food chain [8]. Exposing to mercury results in neurological disorders, damage to central nervous systems, and also negatively affects the kidney and liver [9]. Considering these facts, there should be a proper way to handle this element and remove it from the environment.

Various methods have been used to remove mercury from water and wastewater, including chemical precipitation, ion exchange and membrane methods [10]. Nevertheless, these methods have their own weaknesses including high level of either energy or chemical compound is needed, and most importantly these methods are not able to remove low concentration of mercury from the environment. However, adsorption due mostly to its high performance, recoverability, and reactive ability of adsorbent can be considered as a suitable method in terms of economy [11, 12].

In adsorption process, it is needed to have an adsorbent with wide specific surface. The surface results from the existence of tiny pores at it which the chemical property, area, size and distribution of these pores influence the level of an adsorbent’s specific surface. Different materials, such as fruit shell [13], chitosan [14], marine macroalga [15], bagasse pith [16], furfural [17], and rubber [18] have been applied as adsorbents to remove mercury from aqueous environments.

After the discovery of carbon nanotubes, scientists paid specific attention to them because of their particular efficiency in construction, electricity, chemistry, and physics. Also, these materials have widely been used to produce nano-structured materials, nanocomposites, sensors, and gas adsorption. In 2004, when EPA proposed a research into the environmental application of these materials [19], wide ranges of studies were conducted on these nanotubes which have tiny pores with uniform size and also wide specific surface [20]. Usage of carbon nanotubes was studied to remove pollutants, such as fluoride [21], dichlorobenzene [22], trihalomethanes [23], zinc [24], chromium [25], nickel [26, 27], and cadmium [28] from water and waste water. In this paper, Multi-walled carbon nanotubes) MWCNT_S_ (were used to remove inorganic mercury from aqueous solutions.

## Materials and methods

**Fig. 1:**
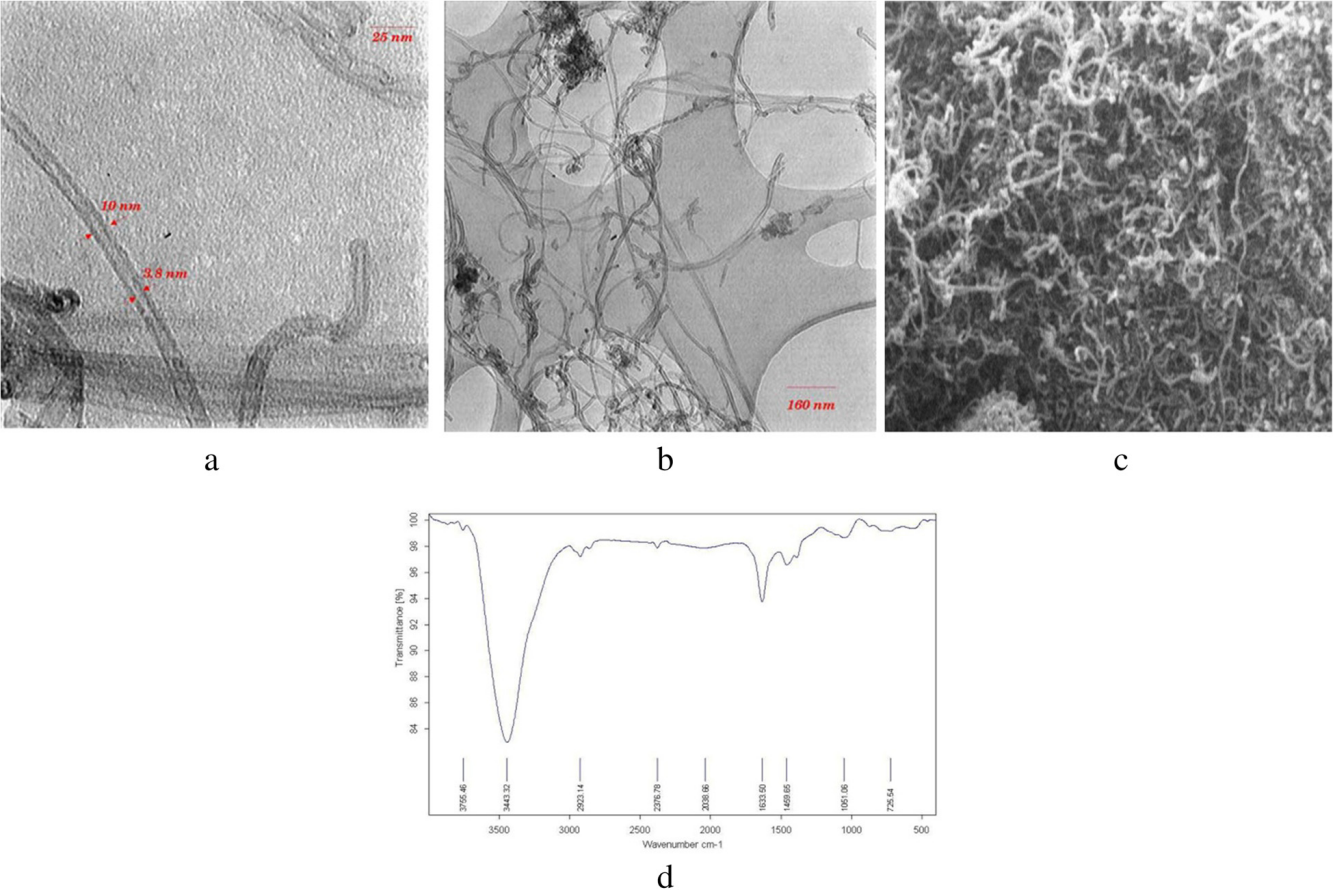
TEM (**a** and **b**), SEM (**c**) and FTIR spectra (**d**) of MWCNTs

Mercury solution was prepared by using HgCl_2_ (Merk) and deionized water. Carbon nanotubes were obtained from research division of Iranian Petroleum Industry. The characteristics of applied nanotubes in this study were as follow: the BET surface area of 270 m^2^/g; diameters of 10–30 nm, respectively; length of 10 μm, and over 95 % purity. Furthermore, the morphology and size of carbon nanotubes were characterized by transmission electron microscope (TEM) and scanning electron microscope (SEM). The surface functional groups of multi-walled carbon nanotubes were detected by Fourier transform infrared spectroscopy (FTIR). Figure 1 shows the TEM, SEM and FTIR images of carbon nanotubes.

Batch reactors with the same volumes of 250 ml were used. The reactors were filled with 100 ml of mercury solution with the concentrations of 0.1, 1 and 10 mg/l. The pH of the solutions were adjusted by nitric acid 0.1 N and NaOH 0.1 N (Merk). After adjusting the pH, the solutions were agitated under the temperature of 25 °C and 150 rpm on an Incubator Shaker (Innova 4340, USA). Thereafter, the solution was passed through 0.2 μm Millipore filter in order to separate the adsorbents from the aqueous solutions. Then, the pH was adjusted and lowered to <2 by using nitric acid. It should be noted that the prepared solutions were kept in glass containers at 4 °C. Besides, Cold vapour inductively coupled plasma optical emission spectrometry (Spectro, Germany) was applied to measure the concentration of mercury. This method has high sensitivity, excellent detection limits, rapid analysis and Easy to use. Also chemical and spectral interference is less than other methods [29]. For the reliable determination of mercury all variables were measured at least twice.

Some variables were considered in previous studies the removal of metal ions from aquatic environments by carbon nano tubes (Table 1). The effects of contact time, primary concentration of the solution, adsorbent’s concentration, ionic strength of the solution, and pH on the process of adsorption were assessed in this research. Freundlich and Langmuir adsorption isotherms were used to study the adsorption isotherm. Also pseudo-first- order and pseudo-second-order kinetics models were applied to determine the adsorption kinetic model.

**Table. 1:** considered variables in previous studies the removal of metal ions from aquatic environments by carbon nanotubes

Metal ion	Variables	Ref	Metal ion	Variables	Ref
zinc	pH, contact time, initial metal ion concentration. Isotherm models	[24]	Zinc	adsorption kinetic, Isotherm models	[30]
Chromium	pH, contact time, agitation speed	[25]	copper	pH, ionic concentration, Isotherm models	[31]
nickel	pH, contact time, initial metal ion concentration, adsorbent’s concentration, Isotherm models	[26]	Lead	pH, contact time, agitation speed, adsorbent’s concentration, adsorption kinetic, Isotherm models	[32]
cadmium	pH, contact time, initial metal ion concentration, temperature, adsorption kinetic, Isotherm models	[28]	Lead	contact time, pH, ionic strength, foreign ions	[33]

The amount of adsorbed Hg^2+^ was calculated through the following equation [24–30]:

1 $$ q=\frac{\left({C}_o-{C}_t\right)\times V}{m} $$

where q denotes the amount of Hg^2+^ adsorbed on adsorbent at any time (mg/g), C_0_ denotes the initial Hg^2+^ concentration (mg/l), C_t_ denotes the concentration of Hg^2+^at any time (mg/l), V denotes the volume of the solution (l) and *m* denotes the adsorbent mass (g).

## Results and discussion

### The surface functional groups of MWCNTs

Figure 1(d) depicts the FTIR spectra of multi-walled carbon nanotubes. This spectrum displays major peaks at 3755, 3443, 2923, 1633, 1459 and 1051 cm^−1^. The peak at 3755 cm^−1^ is associated with free hydroxyl groups. The peak at 3443 cm^−1^ is attributed to the O–H stretch from carboxyl groups (O = C − OH and C − OH). The peak at 2923 cm^−1^ is associated with (CH2). The peak at 1633 cm^−1^ is related to carbonyl groups. The peak at 1459 cm^−1^ is associated with carboxylic acids and phenolic groups (O–H). The peak at 1051 cm^−1^ is attributed to the (C–O). These functional groups can prepare numerous chemical sorption sites on surface of the MWCNTs [24, 28, 30, 32, 33].

### Effect of contact time

**Fig. 2:**
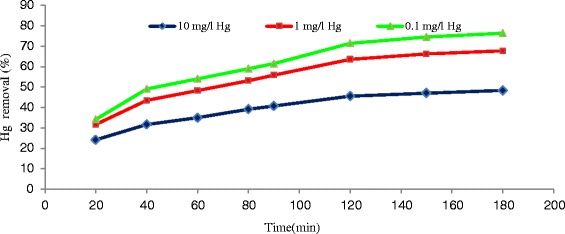
Effect of contact time on the removal of mercury by MWCNT_S_ at different mercury concentrations (adsorbent dose = 0.5 g/l, T = 25 °C, pH =5, agitation speed = 150 rpm)

**Fig. 3:**
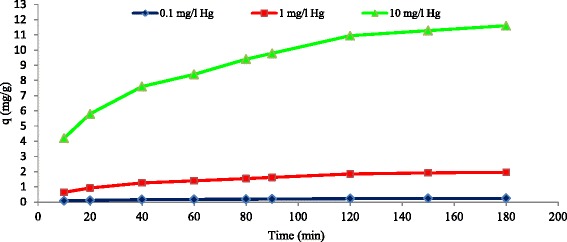
Effect of contact time on the q at different mercury concentrations adsorbed on to MWCNT_S_ (adsorbent dose = 0.5 g/l, T = 25 °C, pH =5, agitation speed = 150 rpm)

As it is indicated in the Fig. 2, the effect of contact time on the level of adsorption of inorganic mercury was evaluated on the surface of multi-walled carbon nanotubes. In this test, the primary concentrations of inorganic mercury were 0.1, 1, and 10 mg/l and the other factors including temperature, pH, concentration of adsorbent, and agitation speed were kept constant. The adsorption of mercury increased with time until it reached equilibrium. The contact time for attainment to equilibrium was 120 min. Also Fig. 2 shows that the increase in the initial concentrations of mercury ions did not affect the equilibrium time. Similar findings have been reported for sorption of Ni^+2^ on carbon nanotubes [26] In addition, the rate of adsorption was reduced with the increase of contact time, which this reduction can be due to the saturation of adsorption points on the carbon nanotubes. ‘*q*’ which is the amount of adsorbed mercury per the weight of carbon nanotubes raised by increase of contact time (Fig. 3).

### Effect of primary concentration

**Fig. 4:**
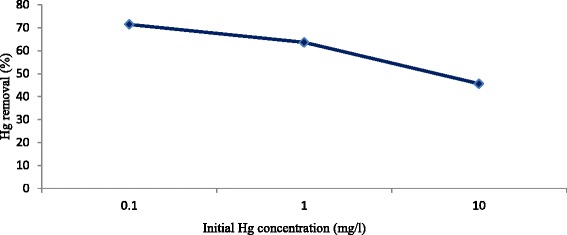
Effect of primary Hg concentration on the removal of mercury by MWCNT_S_ (adsorbent dose = 0.5 g/L, T = 25 °C, pH =5, agitation speed = 150 rpm, contact time = 120 min)

The level of mercury removal in the pH of 5, contact time of 120 min, temperature of 25 °C, agitation speed of 150 rpm, and primary concentration of 0.1, 1, and 10 were 71.4, 63.6 and 45.6 % respectively (Fig. 4). the percentage of mercury removal was declined when the concentration of mercury increased from 0.1 to 10 mg/l. Primary concentration also affects the removal of chrome from aqueous solutions by carbon nanotubes [34]. When the concentration of adsorbent in the solution is constant, the number of adsorbent places is also constant for metallic ions. In this condition, the increase in the number of such ions results in the increase of competence among these ions to be adsorbed and it leads to the decrease of metallic ions’ removal due mainly to electrical repulsive force [34]. It is noteworthy that the increase of primary concentration do not influence the balance time but it significantly affects ‘q’ (Fig. 3). ‘*q*’ raised with increasing of zinc primary concentration in the removal of zinc from aqueous solutions by carbon nanotubes [24].

### Effect of solution’s pH

**Fig. 5:**
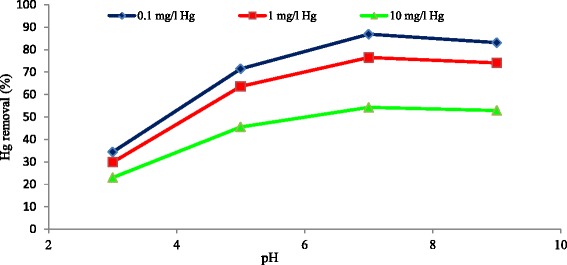
Effect of pH on the removal of mercury by MWCNT_S_ at different mercury concentrations (adsorbent dose = 0.5 g/l, T = 25 °C, contact time = 120 min, agitation speed = 150 rpm)

pH is known as an important and effective parameter on adsorption process. The pH effects not only include the type of ion but also include the properties of adsorbent surface such as surface active groups [35]. As it is shown in the Fig. 5, the effects of pH on the level of mercury’s adsorption on carbon nanotubes in the *conditions* of mercury’s concentrations of 0.1, 1, and 10 mg/l and temperature of 25 °C, contact time of 120 min, adsorbent dosage of 0.5 g/l and agitation speed of 150 rpm were assessed. Based on the results, pH has a significant effect on the process of adsorption and the increase of it raises the capacity of mercury’s adsorption. Besides, the appropriate pH achieved in this study is 7, and below the 5, mercury transforms to Hg^+2^ which competes with H^+^ for the adsorbent places. Hence, at the low pH, the level of adsorption decreases. It is mainly due to this fact that the H^+^ level declines when the pH increases. The results of the present study are in the line with the studies conducted by Gao et al. [36] on the removal of Cu, Cd, Ni, and Zn by carbon nanotubes, and Atieh et al. [32] on the removal of Pb by the same adsorbent.

After the test, the pH was measured again and it was cleared that in the primary pH of upper than 5, the final pH decreases. The release of H^2+^ from the surface of carbon nanotubes into the solution could be the reason of this decrease. In the same contact time, the decrease of final pH was higher for the high concentration of mercury then the low concentration of mercury. When the primary concentration of mercury increases, the amount of adsorption of Hg^+2^ also gets higher by which the rate of H^2+^ release from the adsorbent surface increases and causes to the drop of the pH level of the solution [37]. This process can be the indicator of chemical adsorption of inorganic mercury on the surface of the carbon nanotubes in pH of over 5.

### Effect of adsorbent’s concentration

**Fig. 6:**
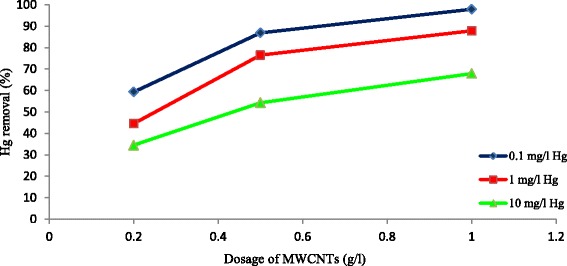
Effect of adsorbent dosage on the removal of mercury by MWCNT_S_ at different mercury concentrations (T = 25 °C, contact time = 120 min, agitation speed = 150 rpm, pH = 7)

The results indicated that the percentage of mercury’s removal increased when the concentration of carbon nanotubes increased from 0.2 to 0.5 and finally to 1 mg/l (Fig. 6). This is mostly due to this reason that the increased concentration of adsorbent makes more active surface [33]. Similar findings have been reported for sorption of Ni^+2^ on carbon nanotubes [27].

### Effect of ionic concentration

**Fig. 7:**
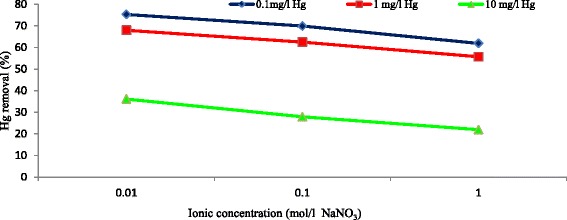
Effect of Ionic concentration on the removal of mercury by MWCNT_S_ at different mercury concentrations (adsorbent dose = 0.5 g/l, T = 25 °C, contact time = 120 min, agitation speed = 150 rpm, pH = 7)

Based on the results, high ionic concentration has negative effect on the removal of mercury (Fig. 7). The reason is probably the impact of ionic concentration on the transfer of Hg^+2^ from the solution to the surface of adsorbent. In a study conducted by Lu and Liu [27], the authors found that the rate of Ni^2+^ adsorption on the surface of carbon nanotubes decreases when the ionic concentration increase.

### Adsorption isotherm

Adsorption isotherms describe the distribution of metal ions between the liquid and solid phase at equilibrium. Adsorption balance of metallic ions is usually studied by the Freundlich and Langmuir adsorption isotherm. Langmuir isotherm is the indicator of active surface adsorption on the homogenous surface, while the Freundlich isotherm is used for heterogeneous surfaces [11]. The linear form of Langmuir and Freundlich equations are as below.

#### Langmuir

2 $$ \frac{{\mathrm{C}}_{\mathrm{e}}}{{\mathrm{q}}_{\mathrm{e}}}=\frac{1}{k{q}_m}+\frac{{\mathrm{C}}_{\mathrm{e}}}{{\mathrm{q}}_{\mathrm{m}}} $$

#### Freundlich

3 $$ \log\ {\mathrm{q}}_{\mathrm{e}} = \log\ {\mathrm{K}}_{\mathrm{F}} + 1/\mathrm{n}\  \log\ {\mathrm{C}}_{\mathrm{e}} $$

Where C_e_ denotes the equilibrium concentration of Hg^+2^ (mg/l), q_e_ denotes the amount adsorbed (mg/g), q_m_ denotes the theoretical saturated adsorption capacity (mg/g), K denotes the Langmuir constant (l/mg). The values of K and q_m_ were calculated by plotting C_e_/q_e_ versus C_e_. K_F_ and n are the Freundlich constants related to adsorption capacity and adsorption intensity, The Freundlich constants n and K_F_ were calculated by plotting log q_e_ versus log C_e_.

The information of the two adsorption isotherms of inorganic mercury on the surface of carbon nanotubes are indicated in Table 2. As can be inferred from the information, the process of adsorption follows both Freundlich and Langmuir isotherms. Li et al. [38] depicted that adsorption of lead on to carbon nanotubes follows both Langmuir and Freundlich equations. Besides, the maximum adsorption capacity obtained from the Langmuir isotherm is 25.641 mg/g. Numerous low cost organic and inorganic adsorbents (e.g. activated carbon) have been explored for mercury removal [39, 40]. Zabihi et al. [41], Di Natale et al. [42], Asasian et al. [43] used different activated carbons to remove mercury that adsorption capacities of activated carbons were different. These amounts were strongly dependent on adsorption conditions such as solution’s pH, adsorbent’s concentration, temperature, ionic concentration and especially initial concentration of mercury [42]. However, activated carbons have disadvantages like weak physical stability, low selectivity for mercury, poor reactive ability of adsorbent and the release of mercury vapor into the atmosphere [39].

**Table. 2:** The parameters of Langmuir and Freundlich isotherm models for the removal of Hg^+2^ by MWCNT_S_

Isotherm models	Parameters	value
Langmuir	q_m_ (mg/g)	25.641
	k (l/mg)	0.565
	R^2^	0.948
Freundlich	K_F_ (l/ g)	0.077
	N	0.7173
	R^2^	0.999

### Adsorption kinetic

The adsorption kinetic model can provide suitable information for designing the removal of pollutants from water and wastewater. In order to assess the adsorption kinetic of inorganic mercury on the surface of carbon nanotubes, the pseudo-first and pseudo-second orders of kinetic equations were applied.

The pseudo first-order equation is written as:

4 $$ \frac{{\mathrm{dq}}_{\mathrm{t}}}{\mathrm{dt}}={\mathrm{k}}_{\mathrm{l}}\left({\mathrm{q}}_{\mathrm{e}}\hbox{-} {\mathrm{q}}_{\mathrm{t}}\right) $$

Where q_t_ is the amount of Hg^+2^ adsorbed at any time (mg/g), q_e_ is the amount of Hg^+2^ adsorbed at equilibrium (mg/g), K_1_ is the adsorption rate constant (1/min).

The integrating for the boundary conditions t = 0 to t = t and q_t_ = 0 to q_t_ = q_e_, gives the linear relationship between the amount of Hg^+2^ adsorbed (q_t_) and time (t).

5 $$ \mathrm{Log}\left({\mathrm{q}}_{\mathrm{e}}\hbox{-} {\mathrm{q}}_{\mathrm{t}}\right)=\mathrm{Log}\;{\mathrm{q}}_{\mathrm{e}}\hbox{-} \frac{{\mathrm{k}}_{\mathrm{l}}}{2.303}\mathrm{t} $$

A straight line log (q_e_ -q_t_) versus t indicates the applicability of the pseudo-first order kinetic model.

The pseudo second-order equation can be expressed as:

6 $$ \frac{{\mathrm{dq}}_{\mathrm{t}}}{\mathrm{dt}}={\mathrm{k}}_2{\left({\mathrm{q}}_{\mathrm{e}}\hbox{-} {\mathrm{q}}_{\mathrm{t}}\right)}^2 $$

Where k_2_ denotes the second-order sorption rate constant (g/mg min). Integrating for the boundary conditions, t = 0 to t = t and q_t_ = 0 to q_t_ = q_e_, gives the following equation:

7 $$ \frac{\mathrm{t}}{{\mathrm{q}}_{\mathrm{t}}}=\frac{1}{{\mathrm{k}}_{{2\mathrm{q}\mathrm{e}}^2}}+\frac{1}{{\mathrm{q}}_{\mathrm{e}}}\mathrm{t} $$

**Fig. 8:**
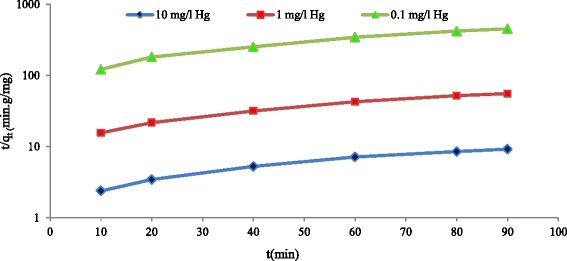
Pseudo- second -order kinetics plot for the removal of Hg^+2^ by MWCNT_S_ at different mercury concentrations (adsorbent dose = 0.5 g/l, T = 25 °C, agitation speed = 150 rpm, pH = 5)

The information regarding these two equations is provided in Fig. 8 and Table 3. The achieved q_e_ from the pseudo-first order adsorption kinetic for the concentration of 0.1, 1, and 10 mg/l are 0.17, 1.40, and 8.1 mg/g, respectively. The corresponding scores for the pseudo-second order adsorption kinetic are 0.24, 1.99, and 11.76 mg/g, respectively, while the experimental q_e_ for the primary concentrations of mercury are 0.23, 1.84, and 10.94, respectively. The process of adsorption of inorganic mercury on the surface of multi-walled carbon nanotubes mostly fits with the second order adsorption kinetic and it is because of this that the q_e_ achieved from the pseudo-second order equations is closer to q_e_ of the base experiments, compared to the pseudo-first order equations. Also, R^2^ conceived from the pseudo-second order adsorption kinetic graph is higher than that of pseudo-first order graph. Previous studies also showed that absorbed cadmium [28], zinc [30], lead [33] and chromium [34] on carbon nanotubes follow pseudo-second order adsorption kinetic.

**Table. 3:** Kinetic parameters for the removal of Hg^+2^ by MWCNT_S_ at different mercury concentrations

Kinetic model	primary Hg^+2^ concentration (mg/l)		
	0.1	1	10
Pseudo-first order			
q_e_, exp (mg/g)	0.23	1.84	10.94
q_e_, cal (mg/g)	0.17	1.40	8.1
K_1_ (1/min)	0.0184	0.0184	0.0207
R^2^	0.99	0.991	0.992
Pseudo-second-order			
q_e_, cal (mg/g)	0.24	1.99	11.76
k_2_ (g/mg min)	.0187	0.022	0.0042
R^2^	0.996	0.997	0.995

Qu et al. [44] reported that nanomaterials have been widely used to remove heavy metals from water due to their large surface area, high reactivity, short intra particle diffusion distance and low temperature modification. In spite of that Tang et al. [45] suggested the reuse and management of the used nanomaterials is an important issue and has not been considered seriously. Only a few relevant studies are available in literature. It would be worthwhile to investigate the reusability of the used nanomaterials.

## Conclusion

Multi-walled carbon nanotubes were assessed as adsorbent to remove inorganic mercury from aqueous solutions. The adsorption rate of Hg^2+^ on the surface of adsorbent is highly affected by pH, and the increase of pH from 3 to 7 increases the percentage of removal. The best contact time is 120 min. Also, the increases of primary concentration of inorganic mercury and ionic concentration solution have negative effect on adsorption process. Finally, the process of adsorption follows both Freundlich and Langmuir isotherms, and the pseudo-second order adsorption kinetic can well describe adsorption process. The present study indicated that Carbon nanotubes have high efficiency in adsorbing of mercury.

## Abbreviations

ICP-OES-EOP: Inductively coupled plasma optical emission spectrometry (End-on-Plasma)
MWCNTS: Multi-walled carbon nanotubes
